# Editorial: Cell death mechanisms in neurodegenerative disorders

**DOI:** 10.3389/fcell.2025.1694173

**Published:** 2025-10-09

**Authors:** Meghana Tare, Beatrice D’Orsi, Amit Singh

**Affiliations:** ^1^ Department of Biological Sciences, Birla Institute of Technology and Science Pilani, Pilani, Rajasthan, India; ^2^ Institute of Neuroscience, Italian National Research Council (CNR), Pisa, Italy; ^3^ Department of Biology, Center for Tissue Regeneration and Engineering at Dayton (TREND), University of Dayton, Dayton, OH, United States

**Keywords:** neurodegeneration, Alzheimer disease, Parkinson’s disease, Huntington (disease), cell death, neuroinflammation, R loop, stroke

## Introduction

Cell death regulation is one of the important features that underlies the health and diseased conditions. All major neurodegenerative and neurological disorders inclusive of cancers and neurodegeneration can widely be attributed to dysregulated cell death mechanisms. Millions of people worldwide suffer from neurological and neurodegenerative disorders such as Alzheimer’s Disease (AD), Parkinson’s Disease (PD), Amylotrophic Lateral Sclerosis (ALS), Huntington’s Disease (HD), Multiple Sclerosis (MS) and Stroke (Sarkar et al., 2016; Narwal et al., 2024). A common underlying feature for all these conditions is neuronal cell death, which has been studied in great detail by researchers for understanding the “cause and the effect” of the symptoms (Singh, 2012). Current understanding gathered from different model organisms, organoid transplants and the *in-vitro* approaches points in the direction that the majority of neurodegenerative disorders are due to proteinopathies, which eventually result in neuronal cell death.

This Research Topic emphasizes on the advancements in understanding the varied mechanisms underlying different types of cell death(s) during the course of neurodegenerative disorders. The Research Topic advances the understanding and raises new questions to aid ongoing research across different model systems. Conservation of genetic machinery and amenability of various models to genetic manipulation helped address these questions in various animal models. We believe that this Research Topic enables researchers to develop detailed understanding of cell death mechanisms, unravel the gaps and missing links and identify the interconnecting pathways to discern neurodegenerative disorders to develop better therapies and management strategies.

## Cell death pathways in neurodegeneration

Studies from invertebrate and mammalian models suggest that neurodegenerative disorders involving protein misfolding and aggregations such as AD, PD and ALS, are characterized by activation of various kinds of cell death pathways including apoptosis, autophagy, ferroptosis and necrosis (Rai and Bergmann, Li et al.). Interestingly, interpreted factors, which are common neurodegeneration cascades from these models are production of ROS, mitochondrial dysfunction and disturbed immune response pathways. There are multiple studies which indicate connections between protein aggregation, ROS production leading to activation of inflammatory pathways involving different types of neuronal and non-neuronal cells (Deshpande et al., 2024).

Abnormal iron homeostasis affects protein aggregations, leading to enhanced ROS production causing ferroptosis in case of PD. Studies from glia-neuron interactions have provided evidence for the role of glial cells in maintenance of iron homeostasis regulating dopaminergic neuronal loss (Li et al.). Activation of glial cells results in activation of Nuclear Factor-Kappa-B (NF-kB) which causes upregulation of inflammatory molecules, thereby causing inflammation in neuronal tissues in conditions of neurodegeneration (Reviewed in Heneka et al., 2025). In addition to apoptosis, autophagy, ferroptosis, necrosis and pyroptosis have also been well documented in conditions of neurodegeneration and neurological conditions such as stroke (Rinald and Troy).

Genetic as well as environmental factors have been extensively studied which can cause neurodegenerative and neurological diseases (Banerjee et al., 2022; Iyer et al., 2024; Yogi et al., 2025). Altered nucleic acid metabolism has also been studied in context to neurodegenerative disorders (Chimata et al., 2024; Singh et al., 2025). Interestingly, three stranded non-canonical DNA-RNA hybrid templates have also been identified to be involved in mediating cellular damage enhancing pathogenesis of several human diseases, including neurodegeneration mediated in ALS, ataxia and spinal muscular atrophy (Liu et al.).

Different kinds of cell death regulate the progression of neurodegenerative and neurological disorders/injuries, it is noteworthy that modeling of different kinds of cell death in different model systems is crucial to develop not only therapeutics, but also early diagnosis. We acknowledge the insights into cell death regulatory pathways developed from models including invertebrate models such as flies up-to mammalian systems, organoid transplants and patient samples.

Articles presented in this section have highlighted various kinds of cell deaths which mediate neuronal loss in different conditions. It is evident that mechanisms involved in mediating neurodegenerative and neurological disorders are multifactorial; possibly due to different mechanisms which mediate neuronal injury and eventual loss.

We believe that this Research Topic will aid in advancing the understanding of the field of neurodegenerative disorders in order to develop better diagnostic and therapeutic measures in future.
